# Current state of clinical ultrasound neuromodulation

**DOI:** 10.3389/fnins.2024.1420255

**Published:** 2024-06-19

**Authors:** Eva Matt, Sonja Radjenovic, Michael Mitterwallner, Roland Beisteiner

**Affiliations:** Functional Brain Diagnostics and Therapy, Department of Neurology, Medical University of Vienna, Vienna, Austria

**Keywords:** transcranial ultrasound, neuromodulation, non-invasive brain stimulation, transcranial pulse stimulation, transcranial focused ultrasound

## Abstract

Unmatched by other non-invasive brain stimulation techniques, transcranial ultrasound (TUS) offers highly focal stimulation not only on the cortical surface but also in deep brain structures. These unique attributes are invaluable in both basic and clinical research and might open new avenues for treating neurological and psychiatric diseases. Here, we provide a concise overview of the expanding volume of clinical investigations in recent years and upcoming research initiatives concerning focused ultrasound neuromodulation. Currently, clinical TUS research addresses a variety of neuropsychiatric conditions, such as pain, dementia, movement disorders, psychiatric conditions, epilepsy, disorders of consciousness, and developmental disorders. As demonstrated in sham-controlled randomized studies, TUS neuromodulation improved cognitive functions and mood, and alleviated symptoms in schizophrenia and autism. Further, preliminary uncontrolled evidence suggests relieved anxiety, enhanced motor functions in movement disorders, reduced epileptic seizure frequency, improved responsiveness in patients with minimally conscious state, as well as pain reduction after neuromodulatory TUS. While constrained by the relatively modest number of investigations, primarily consisting of uncontrolled feasibility trials with small sample sizes, TUS holds encouraging prospects for treating neuropsychiatric disorders. Larger sham-controlled randomized trials, alongside further basic research into the mechanisms of action and optimal sonication parameters, are inevitably needed to unfold the full potential of TUS neuromodulation.

## 1 Introduction

With its unique capability to non-invasively reach deep brain areas at unparalleled precision, transcranial ultrasound (TUS) applications have attracted increasing interest in basic and clinical research. Depending on focal energy levels, TUS can be administered to achieve highly focal tissue ablation through high-intensity focused ultrasound or for neuromodulation using low-intensity ultrasound which avoids morphological destructions (Beisteiner and Lozano, 2020). Furthermore, concomitant use of TUS and intravenously administered microbubbles has been evaluated for transiently opening the blood–brain-barrier, for example to deliver therapeutic agents (Meng et al., 2019), to clear amyloid-beta accumulations in Alzheimer’s disease (AD, Rezai et al., 2020; Jeong et al., 2022) or to enhance neuromodulatory effects (Jeong et al., 2021). Non-invasive TUS neuromodulation has been investigated using different techniques, starting with unfocussed diagnostic ultrasound systems (Hameroff et al., 2013; Nicodemus et al., 2019), advancing to unnavigated focal applications (e.g., Lohse-Busch et al., 2014; Wang et al., 2022), and resulting in the current state-of-the-art of neuronavigated focal applications, allowing real-time positioning of the ultrasound beam based on individual brain anatomy.

**Figure 1:**
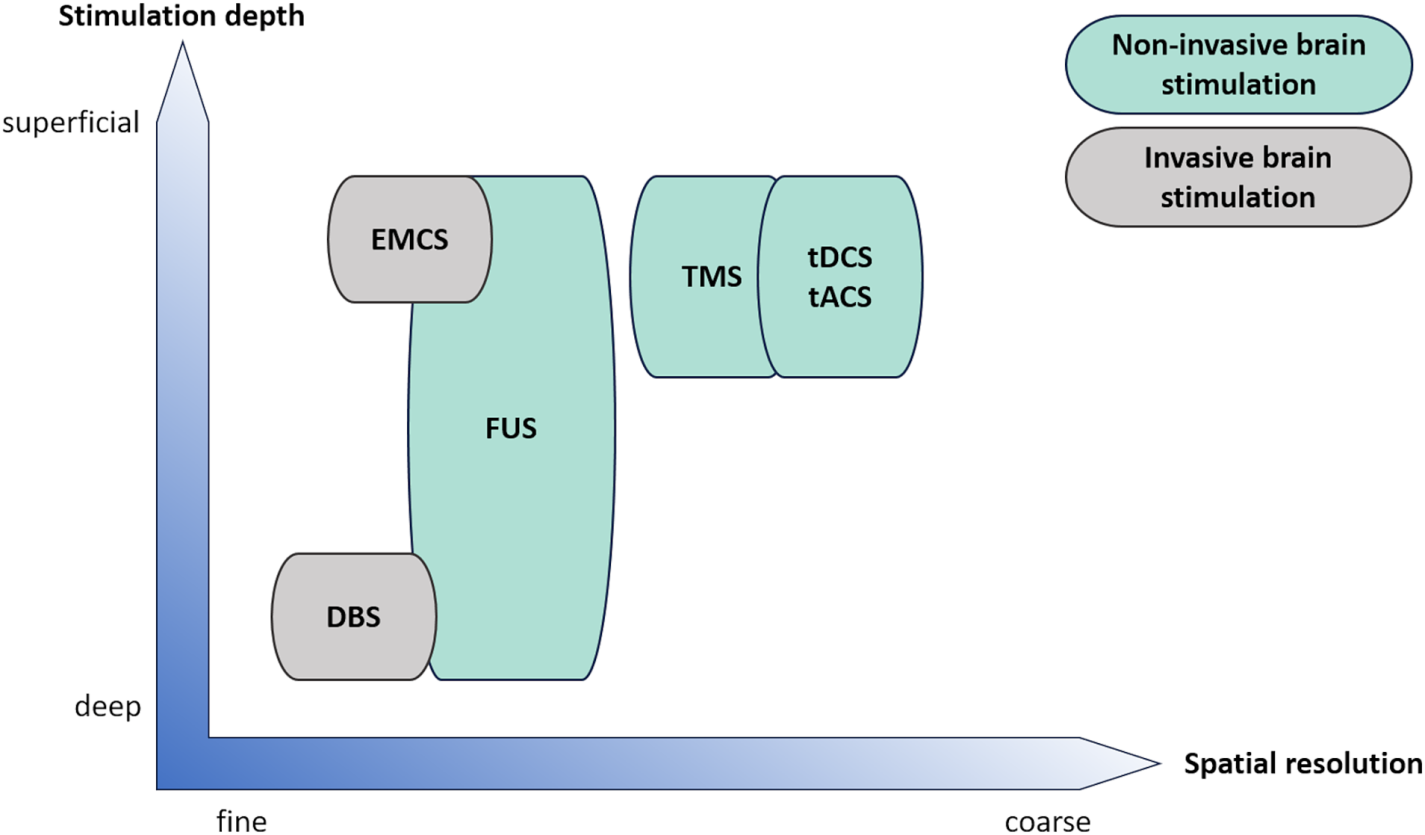
Schematic comparison of invasive (grey) and non-invasive (green) clinical brain stimulation techniques regarding spatial resolution and stimulation depth. DBS, deep brain stimulation; EMCS, extradural motor cortex stimulation; FUS, focused ultrasound; tACS, transcranial alternating current stimulation; tDCS, transcranial direct current stimulation; TMS, transcranial magnetic stimulation.

Two classes of technologies are currently available for navigated focal stimulation, low-intensity transcranial focused ultrasound (e.g., tFUS, LIFUS) and transcranial pulse stimulation (TPS). While tFUS is administered in intermittent trains of ultrasound pulses using sinus tones, TPS applies ultrashort (3 μs) pressure pulses with a range of different frequencies which are repeated at 1 to 8 Hz (for review see Beisteiner et al., 2023). Both techniques result in an elongated elliptical ultrasound beam with an axial resolution of approximately 4 cm and a transversal resolution of approximately 4 mm full width at half maximum for typical carrier frequencies of 500 kHz for tFUS and 250 kHz for TPS (Beisteiner et al., 2019; Truong et al., 2022). This spatial resolution surpasses non-invasive brain stimulation methods based on electromagnetic fields, such as transcranial magnetic stimulation (TMS) or transcranial direct current stimulation (tDCS), by far (Figure 1). TPS and certain tFUS systems are currently approved for research purposes (Investigational Device Exemption by the FDA), and TPS is authorized for the treatment of AD (CE certification).

The growing number of clinical investigations on focused ultrasound in neurological and psychiatric disorders in recent years underscores the necessity for an update of current evidence and forthcoming research initiatives planned in the field. This includes investigations concerning pain, dementia, psychiatric disorders, movement disorders, epilepsy, disorders of consciousness, and other disorders, as detailed in the subsequent section. A critical synthesis of existing evidence, limitations, and future trajectories for TUS are provided in the Discussion.

## 2 Evidence in clinical populations

### 2.1 Pain

The first report of TUS neuromodulation in humans was provided by Hameroff et al. (2013) who applied an unfocused diagnostic ultrasound device in 31 chronic pain patients in a sham-controlled crossover trial. Compared to sham, verum ultrasound application to the right posterior frontal cortex led to significant improvements in mood and a trend for pain reduction (see Table 1 for details). Focused tFUS directed at the dorsal anterior cingulate cortex reduced pain ratings for up to 4 weeks as found in an open-label study involving 11 patients with chronic neuropathic pain (Shin et al., 2023).

**Table 1:** Publications on clinical ultrasound neuromodulation.

Studies	Sample	Study characteristics	Systems	Targets	Main findings
Pain					
Hameroff et al. (2013)	31 patients with chronic pain	Sham-controlled, cross-over, double-blind	Diagnostic system (GE-LOGIQe)	Posterior right frontal cortex	Trend for pain reduction, mood improvement (VAMS)
Shin et al. (2023)	11 patients with chronic neuropathic pain	Open-label, uncontrolled	Focused navigated system (NS-US100, Neurosona Corporation)	Dorsal anterior cingulate cortex	Reduced pain ratings
Dementia and other cognitive impairments					
Beisteiner et al. (2019)	35 AD patients	Open-label, uncontrolled, multicentric	Focused navigated transcranial pulse system (TPS, Storz Medical AG)	DLPFC, DMN	Improved cognition scores (CERAD) over 3 months, reduced depressive symptoms (BDI-II, GDS). Upregulation in task based and resting state fMRI
Nicodemus et al. (2019)	11 AD patients, 11 PD patients	Open-label, uncontrolled	Diagnostic system (DWL Doppler Box X)	Hippocampus (AD), substantia nigra (PD)	Majority of patients showed improved clinical sores (RBANS, MoCA) and stable fine (9-HPT) and gross motor (T25-FW) functions. MRI Perfusion increase in 2 patients.
Popescu et al. (2021)	17 AD patients	Follow-up study, open-label, uncontrolled	Focused navigated transcranial pulse system (TPS, Storz Medical AG)	DLPFC, DMN	Neuropsychological changes (CERAD) were correlated with cortical atrophy changes after TPS.
Dörl et al. (2022)	18 AD patients	Follow-up study; open-label, uncontrolled	Focused navigated transcranial pulse system (TPS, Storz Medical AG)	DLPFC, DMN	Correlation between visuo-constructive score changes and functional connectivity and in the untargeted visuo-constructive network.
Cont et al. (2022)	11 AD patients	Open-label, retrospective, uncontrolled	Focused navigated transcranial pulse system (TPS, Storz Medical AG)	DLPFC, DMN, temporal cortex	Improved cognition scores (ADAS, ADAS-Cog) immediately post treatment
Shimokawa et al. (2022)	15 AD patients	Sham-controlled, parallel-group, double-blind	Custom diffusion type system, LIPUS	Global stimulation	Tendency for better cognitive outcome (ADAS-Cog) with verum stimulation
Wang et al. (2022)	60 patients with post-stroke cognitive impairment	Sham-controlled, parallel-group, double-blind	Focused system (Shengxiang Technology)	5 probes on forehead	Improvements in cognition scores (MMSE, MoCA), Barthel Score. Increased EEG P300 latency and amplitude, elevated BDNF levels.
Fong et al. (2023)	19 patients with mild neurocognitive disorder	Open-label, uncontrolled	Focused navigated transcranial pulse system (TPS, Storz Medical AG)	Global stimulation	Improved cognitive scores (MoCA, Verbal Fluency, Stroop interference) and IADL. No change in serum BDNF level.
Psychiatric disorders					
Reznik et al. (2020)	24 participants with depression	Randomized, sham-controlled, parallel-group, double-blind	Focused system (Neurotrek U+, Neurotrek Inc.)	F8 (right frontal EEG position)	No changes in depression (BDI-II) or anxiety (OASIS), trait worry decreased (PSWQ) and global affect improved (VAMS)
Matt et al. (2022a)	18 AD patients	Follow-up study; open-label, uncontrolled	Focused navigated transcranial pulse system (TPS, Storz Medical AG)	DLPFC, DMN	Reduction of depressive symptoms (BDI-II). Normalization of the functional connectivity between the salience network and the ventromedial network
Cheung et al. (2023a)	30 patients with major depressive disorder	Waiting list control group, single-blind	Focused navigated transcranial pulse system (TPS, Storz Medical AG)	Left DLPFC	Significant improvements of depressive symptoms (HDRS-17), anhedonia (SHAPS), IADLs, and cognitive performance (MoCA, digit span, trail making test).
Mahdavi et al. (2023)	25 patients with generalized anxiety disorder	Open-label, uncontrolled	Focused navigated system (Brainsonix)	Right amygdala	Decrease in anxiety (HAM, BAI)
Zhai et al. (2023)	26 patients with schizophrenia	Randomized, sham-controlled, parallel-group, double-blind	Custom focused navigated system	Left DLPFC	Alleviation of negative symptoms (SANS) and general schizophrenia symptoms (PANSS), enhanced cognitive performance (CPT)
Riis et al. (2023)	1 patient with treatment resistant depression	Open-label, uncontrolled	Focused navigated system (Diadem)	Subcallosal cingulate cortex	Reduction of the HDRS-6 score of 11 to 0
Movement disorders					
Deveney et al. (2024)	10 patients with essential tremor	Open-label, uncontrolled	Focused navigated system (Brainsonix)	VIM (thalamus)	Reduced essential tremor (TETRAS) in all patients
Osou et al. (2023)	20 PD patients	Open-label, retrospective, uncontrolled	Focused navigated transcranial pulse system (TPS, Storz Medical AG)	Motor network	Significant improvement in motor symptoms (UPDRS-III)
Samuel et al. (2023)	10 PD patients	Sham-controlled, cross-over, double-blind	Custom focused navigated system	Motor cortex	Increased motor cortex excitability but no changes in motor symptoms (UPDRS-III)
Grippe et al. (2024)	20 PD patients (on and off medications), 17 controls	Open-label, case–control study	Custom focused system (Sonic Concepts)	Motor cortex	Increased motor-evoked potential amplitude and reduced bradykinesia in patients on dopaminergic therapy
Bancel et al. (2024)	9 patients with essential tremor	Open-label, uncontrolled	Focused navigated system (Insightec Exablate Neuro)	Thalamus (VIM and dentato-rubro-thalamic tract)	Tremor reduction in 5 patients
Riis et al. (2024)	3 patients with essential tremor	Open-label, uncontrolled	Focused navigated system (Diadem)	VIM (thalamus)	Reduction in the tremor amplitude in 2 patients
Epilepsy					
Brinker et al. (2020)	1 DRE patient	Open-label, uncontrolled	Custom focused navigated system	Hippocampus	No outcomes analyzed
Stern et al. (2021)	8 DRE patients	Open-label, uncontrolled	Focused navigated system (BX Pulsar, Brainsonix)	Anterior medial temporal lobe	Slight performance decrease in a verbal memory test (RAVLT). No evidence of histological changes due to tFUS
Lee et al. (2022)	6 DRE patients	Open-label, uncontrolled	Focused navigated multichannel system (NaviFUS corporation)	Individual seizure onset zone	Seizures decreased in 2 patients and increased in 1 patient. Spectral power of stereo-EEG changed during tFUS
Bubrick et al. (2024)	6 DRE patients	Open-label, uncontrolled	Custom focused navigated system	Hippocampus	Significant seizure frequency reduction in 5 patients
Disorders of consciousness					
Lohse-Busch et al. (2014)	5 patients with chronic unresponsive wakefulness	Open-label, uncontrolled	Focused transcranial pulse system (TPS precursor, Storz Medical AG)	Global stimulation	Coma scores (KRS, GCS) improved
Monti et al. (2016)	1 patient with acute post-traumatic DOC	Open-label, uncontrolled	Focused navigated system (Brainsonix)	Thalamus	Recovery from brain injury
Cain et al. (2021)	3 patients with chronic DOC	Open-label, uncontrolled	Focused navigated system (Brainsonix)	Left central thalamus	2 patients showed improved responsiveness (CRS-R)
Cain et al. (2022)	11 patients with acute DOC	Open-label, uncontrolled	Focused navigated system (Brainsonix)	Central thalamus	Significant improvements in coma recovery scale (CRS-R). Correlation between recovery and fMRI connectivity changes
Other disorders					
Cheung et al. (2023b)	34 ASD patients	Sham-controlled, parallel-group, double-blind	Focused navigated transcranial pulse system (TPS, Storz Medical AG)	Right temporoparietal junction	Significant improvement in the severity of clinical symptoms (CARS, CGI)

AD, Alzheimer‘s disease; ADAS, Alzheimer‘s Disease Assessment Scale; ASD, Autism spectrum disorder; BAI, Beck Anxiety Inventory; BDI-II, Beck Depression Inventory; BDNF, brain derived neurotrophic factor; CARS, Childhood Autism Rating Scale; CERAD, Consortium to Establish a Registry for Alzheimer’s Disease Test; CGI, Clinical Global Impression; CPT, Continuous Performance Test; CRS-R, Coma Recovery Scale Revised; DLPFC, dorsolateral prefrontal cortex; DMN, default mode network; DOC, disorder of consciousness; DRE, drug resistant epilepsy; EEG, electroencephalogram; GCS, Glasgow Coma Scale; GDS, Geriatric Depression Scale; HAM, Hamilton Anxiety Inventory; HDRS, Hamilton depression rating scale; IADL, instrumental activities of daily living; KRS, German Coma Remission Scale; MMSE, Mini-Mental State Examination; MoCA, Montreal Cognitive Assessment; MRI, magnetic resonance imaging; OASIS, Overall Anxiety Severity and Impairment Scale; PANSS, Positive and Negative Syndrome Scale; PD, Parkinson’s disease; PSWQ, Penn State Worry Questionnaire; RAVLT, Rey Auditory Verbal Learning Test; RBANS, Repeatable Battery for Assessment of Neuropsychological Status; SANS, Scale for the Assessment of Negative Symptoms; SHAPS, Snaith–Hamilton pleasure scale; T25-FW, Timed 25-Foot Walk; TETRAS, Essential Tremor Rating Scale scores; TPS, Transcranial pulse stimulation; UPDRS, Unified Parkinson’s Disease Rating Scale; VAMS, Visual Analogue Mood Scales; VIM, ventral intermediate nucleus.

Further, sham-controlled investigations in healthy participants support antinociceptive effects of tFUS. Badran et al. (2020) found decreased sensitization to thermal pain in 19 healthy participants following tFUS targeting the anterior thalamus for active compared to sham stimulation. Relative to sham, verum tFUS of the posterior insula resulted in reduced heat pain ratings and affected early EEG components, whereas stimulation of the anterior insula influenced the heart rate variability and later EEG amplitudes (Legon et al., 2024).

Encouraged by these findings, several clinical trials are planned in neuropathic pain using tFUS only (NCT03111277, NCT04485208, JPRN-jRCTs052230116, Table 2) or in combination with tDCS (diabetic neuropathic pain, NCT03625752). This combination is also used for pain in the context of carpal tunnel syndrome (NCT04206215), osteoarthritis (NCT02723929,) and opiate use disorder with chronic pain (NCT04379115). In addition, tFUS applications are currently investigated in trigeminal neuralgia (NCT04579692) and generalized chronic pain (NCT05674903).

**Table 2:** Completed (but not yet published), ongoing, and prospective clinical studies on transcranial ultrasound neuromodulation.

ID	Institute	Diagnosis	Method	Design	*N*	Status
Pain						
NCT04485208	Neurological Associates of West Los Angeles (USA)	Neuropathic pain	tFUS	Open-label	40	Ongoing
NCT04579692	University of Maryland (Baltimore, USA)	Trigeminal Neuralgia	tFUS	Open-label	10	Ongoing
NCT02723929	Spaulding Rehabilitation Hospital (USA)	Pain perception in osteoarthritis	tFUS, tDCS	RCT, parallel, triple-blind	64	Ongoing
NCT04206215	Spaulding Rehabilitation Hospital (USA)	Carpal Tunnel Syndrome (pain)	tFUS, tDCS	RCT, parallel, triple-blind	95	Ongoing
NCT05674903	University of Utah (USA)	Generalized chronic pain	tFUS	RCT, crossover, triple-blind	40	Ongoing
NCT04379115	Case Western Reserve University (Ohio, USA)	Opiate use disorder with chronic pain	tFUS, tDCS	RCT, parallel, triple-blind	30	Ongoing
NCT03625752	Case Western Reserve University (Ohio, USA)	Diabetic neuropathic pain	tFUS, tDCS	RCT, parallel, triple-blind	60	Ongoing
JPRN-jRCTs052230116 (JPRN register)	Osaka University Hospital (Japan)	Neuropathic pain	tFUS	RCT, crossover, single-blind	24	Ongoing
Dementia and other cognitive impairments						
NCT03896698	National Yang Ming University (Taiwan)	AD	tFUS	RCT, parallel, quadruple-blind	10	Completed
NCT03770182	Medical University of Vienna (Austria)	AD	TPS	RCT, crossover, double-blind	60	Completed
NCT04250376	Neurological Associates of West Los Angeles (USA)	MCI, dementia	tFUS	Open-label	100	Ongoing
NCT06135051	University of Utah (USA)	MCI, AD	tFUS	RCT, crossover, triple-blind	40	Ongoing
NCT05417555	University of California (USA)	MCI, AD	tFUS	RCT, parallel, double-blind	144	Ongoing
NCT05499429	Xuanwu Hospital, Beijing (China)	Lewy body dementia	tFUS	RCT, parallel, triple-blind	20	Ongoing
NCT05331560	The University of Hong Kong (China)	Mild neurocognitive disorder	TPS	Open-label	20	Ongoing
NCT05602467	The University of Hong Kong (China)	Mild neurocognitive disorder	TPS	Open-label	22	Ongoing
NCT05762926	University of Sao Paulo General Hospital (Brazil)	AD	TPS	RCT, parallel, double-blind	50	Ongoing
DRKS00033282 (German Clinical Trials Register)	Wahrendorff Klinikum (Germany)	AD	TPS	Open-label	100	Ongoing
NCT06313944	Heinrich-Heine University (Duesseldorf, Germany)	AD	TPS	Open-label	100	Planned for 2024
NCT05910619	University of Florida (USA)	Healthy controls and mild AD	TPS	RCT sham-controlled, parallel, triple-blind	20	Planned for 2024
Psychiatric disorders						
NCT02685488	University of Arizona (USA)	Depression	tFUS	RCT, parallel, double-blind	26	Completed
NCT04405791	Gangnam Severance Hospital (South Korea)	Depression	tFUS	RCT, parallel, double-blind	30	Completed
NCT06085950	Centre Hospitalier St Anne (France)	Depression	tFUS	Open-label	10	Ongoing
NCT06320028	University of Arizona (USA)	Depression	tFUS	Open-label	20	Ongoing
NCT05551585	The Hong Kong Polytechnic University	Depression	TPS	Open-label	80	Ongoing
NCT04250441	Neurological Associates of West Los Angeles (USA)	Depression, Anxiety	tFUS	Open-label	100	Ongoing
NCT05259306	NYU Langone Health (USA)	Schizophrenia	tFUS	Open-label	3	Ongoing
NCT05985993	Shanghai Mental Health Center (China)	Schizophrenia	tFUS	RCT, parallel, double-blind	102	Ongoing
ChiCTR2300079134 (ChiCTR register)	Chaohu Hospital of Anhui Medical University (China)	Anxiety	tFUS	RCT, parallel	60	Ongoing
NCT05301036	University of Utah (USA)	Depression / bipolar disorder	tFUS	RCT, crossover, triple-blind	20	Ongoing
NCT04775875	Neurological Associates of West Los Angeles (USA)	Obsessive-compulsive disorder	tFUS	Open-label	30	Ongoing
NCT06273904	Medical University of South Carolina (USA)	Anxiety	tFUS	Open-label	40	Planned for 2024
						
NCT06249711	University of Utah (USA)	Food addiction	tFUS	RCT, parallel, triple-blind	40	Planned for 2024
NCT06135064	University of Utah (USA)	Post-traumatic stress disorder	tFUS	RCT, crossover, triple-blind	20	Planned for 2024
Movement disorders						
NCT04333511	Medical University of Vienna (Austria)	PD	TPS	RCT, crossover, double-blind	30	Completed
NCT05475340	Neurological Associates of West Los Angeles (USA)	PD, essential tremor	tFUS	Open-label	50	Ongoing
NCT06232629	University Health Network (Toronto, Canada)	PD	tFUS	RCT, crossover, double-blind	10	Ongoing
NCT03981055	Spaulding Rehabilitation Hospital (USA)	PD	tFUS, tDCS, physical therapy	RCT, parallel, triple-blind	40	Ongoing
Epilepsy						
NCT06292494	Taipei Veterans General Hospital (Taiwan)	Epilepsy	tFUS	Open-label	20	Ongoing
						
NCT04999046	NaviFUS Corporation	Epilepsy	tFUS	RCT, crossover, single-blind	12	Ongoing
ChiCTR2300077707 (ChiCTR register)	West China Hospital, Sichuan University (China)	Epilepsy	tFUS	Open-label	10	Ongoing
Other Disorders						
NCT04497363	Neurological Associates of West Los Angeles (USA)	ADHD	tFUS	Open-label	100	Ongoing
NCT05422274	The Hong Kong Polytechnic University (China)	ADHD	TPS	RCT, parallel, double-blind	30	Ongoing
NCT05408793	The Hong Kong Polytechnic University (China)	Autism Spectrum Disorder	TPS	RCT, parallel, double-blind	36	Ongoing
NCT06178952	Medical University of Vienna (Austria)	Post-Covid-19	TPS	RCT; parallel, quadruple-blind, multicenter	120	Ongoing
NCT04770350	Neurological Associates of West Los Angeles (USA)	Age-related frailty	tFUS	Open-label	50	Ongoing
NCT04877184	Cheng-Hsin General Hospital (Taiwan)	Stroke	tFUS	Non-randomized, parallel, double-blind	20	Ongoing

Source: ClinicalTrails.gov, unless otherwise specified.

AD, Alzheimer’s disease; ADHD, attention deficit hyperactivity disorder; MCI, mild cognitive impairment; PD, Parkinson’s disease; RCT, randomized controlled trial; tDCS, transcranial direct current stimulation; tFUS, transcranial focused ultrasound; TPS, transcranial pulse stimulation.

### 2.2 Dementia and other cognitive disorders

Previous findings on TUS regarding reduction of AD-associated amyloid-β depositions and memory improvement in animal models (e.g., Leinenga and Götz, 2015; Eguchi et al., 2018; Bobola et al., 2020), as well as studies on safety and feasibility in healthy participants (e.g., Beisteiner et al., 2019), imply a high potential for therapeutic effects in dementia and other cognitive disorders.

In an open-label study, Nicodemus et al. (2019) directed a diagnostic ultrasound system at the hippocampus in 11 AD patients and at the substantia nigra in 11 patients with Parkinson’s disease (PD) during sleep (see Section 2.3. for motor function evaluation). As reported for the combined AD and PD sample, more than half of the individuals improved in at least one cognitive score (see Table 1 for details). In two patients arterial spin labelling was applied demonstrating increased relative blood flow perfusion after sonication. However, employing identical outcome measures for both AD and PD patients, despite different stimulation targets and the variability of sleep induction—natural or pharmacological—raises questions regarding comparability and interpretation of the findings.

The first clinical investigation with a focused navigated state-of-the-art system was reported by Beisteiner et al. (2019) and this study investigated therapeutic effects in AD. The multicentric open-label study included 35 AD patients who received repeated TPS applications of the dorsolateral prefrontal cortex (DLPFC) and the default mode network. Improved cognitive abilities, particularly in memory and language domains, following TPS interventions persisted up to 3 months and were related to functional upregulation of memory networks. Cognitive enhancements were observed to correlate with cortical atrophy, as evidenced in a subset comprising 17 subjects (Popescu et al., 2021). However, visuo-constructive skills deteriorated after TPS which was associated with a decrease in global efficiency of the visuo-constructive network. As these areas were not targeted by TPS, findings were interpreted as reflecting disease progression within untreated networks, compatible with the high targeting specificity of TUS (Dörl et al., 2022). Using the same technique as Beisteiner et al. (2019), but expanding target regions to the temporal cortex, Cont and colleagues investigated clinical effectiveness of TPS in a retrospective analysis of 11 AD patients and found cognitive enhancements immediately after sonication (Cont et al., 2022). Another open-label TPS study reported a tendency for cognitive improvement in 10 AD patients, along with significantly alleviated neuropsychiatric symptoms 30 days after repeated TPS administration (Shinzato et al., 2024). Further, TPS was reported to improve global cognition, verbal fluency, executive functions, and activities of daily living in an open-label study in 19 older adults with mild neurocognitive disorder (Fong et al., 2023). However, no significant change in the serum level of the brain-derived neurotropic factor (BDNF) was observed here.

Using a diffusion type system adjusted to whole brain low-intensity pulsed sonication, Shimokawa et al. (2022) performed a small sham controlled trial in 22 AD patients, with 10 patients receiving verum sonication and 5 receiving sham stimulation. After more than a year, participants in the treatment group remained cognitively stable while cognitive functions declined in individuals receiving sham. Yet, the difference between groups failed to pass statistical significance, possibly due to the small sample size.

Concerning other cognitive disorders, a randomized sham-controlled trial on post-stroke cognitive impairment included 60 patients who received tFUS treatment of the forehead and cognitive rehabilitation training (Wang et al., 2022). The results depicted significantly higher improvements in cognition, Barthel Score, increased EEG P300 amplitude and latency, and elevated BDNF levels in the verum group.

In conclusion, several open-label studies indicated the potential of TPS and tFUS to improve cognitive deficiencies, as partly supported by functional imaging findings. Larger randomized controlled trials (RCTs) are certainly needed to elucidate the true effect of TUS compared to sham stimulation, as addressed in current and prospective research projects (TPS: NCT03770182, NCT05762926, NCT05910619; tFUS: NCT03896698, NCT06135051, NCT05417555, NCT05499429, see Table 2 for details).

### 2.3 Psychiatric disorders

Earlier research indicated the potential of non-invasive ultrasound applications to ameliorate behaviors related to depression, fear, and anxiety in animal models (Zhang et al., 2021; Yi et al., 2022; Lee et al., 2024), as well as the capacity to enhance global affect and modulate resting state functional connectivity in healthy subjects (Sanguinetti et al., 2020).

In an RCT, 24 participants with mild to moderate depression were treated with tFUS directed at right fronto-temporal areas (Reznik et al., 2020). Despite the absence of significant reductions in depression and anxiety scores, notable improvements were observed in worrying and global affect after the stimulation. Sonication of the left DLPFC using TPS induced a significant symptom reduction in 30 individuals with depression when compared to a waitlist-control group (Cheung et al., 2023a). Additional improvements were noted in daily functioning as well as anhedonia, and effects remained stable even three months after the treatment conclusion. A durable remission was also reported for a case with severe treatment-resistant depression following sonication of the subcallosal cingulate cortex (Riis et al., 2023).

Additionally, depression scores were assessed within the context of other medical conditions (see corresponding sections for details). Regarding AD cohorts, investigations utilizing TPS showed a reduction of depressive symptoms post-treatment (Cont et al., 2022) and up to 3 months (Beisteiner et al., 2019). A follow-up investigation in a subsample of Beisteiner et al. (2019) revealed that depressive alleviation was associated with normalization of functional connectivity between the salience network and the ventromedial network in AD (Matt et al., 2022a). Conversely, in individuals with mild neurocognitive disorders, there was no mitigation of depressive symptoms after TPS (Fong et al., 2023). Nevertheless, patients suffering from chronic pain showed subjective mood improvements after sonication using an unfocused diagnostic device (Hameroff et al., 2013).

Recently, an open-label pilot study was conducted to investigate the efficacy of tFUS in treating treatment-refractory generalized anxiety disorder (Mahdavi et al., 2023). Anxiety symptoms were significantly reduced following tFUS directed towards the right amygdala in a cohort of 25 patients. In schizophrenia, repetitive tFUS was applied to the left DLPFC in a pilot study including 26 patients resulting in significant improvements of negative and general schizophrenia symptoms in the verum tFUS group only (Zhai et al., 2023).

Consistently, TUS neuromodulation has been found to alleviate depressive symptoms and improve mood, even in non-psychiatric samples and with considerably different stimulation sites. However, sham-controlled evidence is still scarce, stressing the need for more randomized sham-controlled TUS trials in psychiatric disorders, as planned for depression (NCT02685488, NCT04405791, NCT05301036), schizophrenia (NCT05985993), anxiety (ChiCTR2300079134), food addiction (NCT06249711), and post-traumatic stress disorder (NCT06135064).

### 2.4 Movement disorders

Studies on cortical excitability of the motor cortex and increase in dopamine release in animals (e.g., Zhou et al., 2019; Wang et al., 2020; Xu et al., 2020) and healthy subjects (e.g., Gibson et al., 2018; Legon et al., 2018; Matt et al., 2022b; Bao et al., 2024) suggest a potential benefit of TUS in movement disorders.

In the aforementioned study by Nicodemus et al. (2019, see Section 2.1), no significant alterations after unfocused sonication were found in AD and PD patients regarding fine and gross motor functions (Nicodemus et al., 2019). Potentially, stimulation using a diagnostic US device was not target-specific enough to elicit measurable changes. Personalized focal TPS treatment of cortical motor areas led to a significant improvement in motor symptoms in a retrospective clinical data analysis including 20 PD patients (Osou et al., 2023). Theta-burst TUS stimulation of the motor cortex increased the amplitude of motor evoked potentials (MEP) and improved bradykinesia in 20 PD patients *on* but not *off* medication, stressing the impact of dopaminergic therapy for treatment responses (Grippe et al., 2024). In a randomized controlled crossover study applying accelerated theta-burst tFUS to the primary motor cortex in 10 PD patients, reduced pathological motor scores were reported as well, but changes after the treatment were not different between verum and sham stimulation (Samuel et al., 2023). Yet, a significantly increased MEP amplitude was observed for verum vs. sham sonication.

In essential tremor, three open-label studies using tFUS to target the ventral intermediate nucleus of the thalamus showed tremor reductions in the majority of the patients (Bancel et al., 2024; Deveney et al., 2024; Riis et al., 2024).

Evidence for clinical benefits of TUS in movement disorders is currently limited to a few, mostly uncontrolled, studies. The only sham-controlled study so far failed to find a significant clinical difference between verum and sham tFUS, possibly due to the small sample size. Particularly in movement disorders such as PD, which is linked to a dopaminergic deficiency, placebo effects are prominent due to activation of dopaminergic reward system triggered by anticipated symptom relief (Osou et al., 2023). Larger, sham-controlled trials with comprehensive assessments of clinical, behavioral, electrophysiological and imaging outcomes are desirable, as planned in current clinical trials (NCT04333511, NCT06232629, NCT03981055).

### 2.5 Epilepsy

Motivated by findings in animal epilepsy models (Min et al., 2011; Hakimova et al., 2015; Fomenko et al., 2020) and reports of decreased EEG potentials in humans (e.g., Legon et al., 2014), tFUS has been discussed as a non-invasive technique to suppress epileptiform activity.

Stern et al. investigated the effects of tFUS in eight patients with medication-resistant temporal lobe epilepsy (Stern et al., 2021). Excitatory and inhibitory tFUS was directed towards the anterior mesiotemporal lobe, a region that was subsequently resected in a prescheduled surgery. No histopathological changes were found causally related to tFUS, but neuropsychological tests indicated a reduction in verbal memory functions in a subgroup of four patients.

In another open-label study, tFUS was administered at the individual seizure onset zone in six patients suffering from drug-resistant epilepsy (Lee et al., 2022). Stereo-EEG revealed significant alterations in the spectral power at the targeted electrodes during the stimulation. The frequency of seizures in the subsequent days decreased in two patients, but another patient experienced more frequent subclinical seizures. One patient exhibited impaired memory and naming capabilities that normalized after 3 weeks.

Bubrick et al. (2024) reported a pilot safety trial administering a custom tFUS device, as introduced by Brinker et al. (2020), targeted to the hippocampus in six patients with drug-resistant epilepsy. After six tFUS sessions with scaled intensity, five patients experienced a significantly reduced seizure frequency which persisted for several months thereafter. Resting state fMRI showed a functional connectivity enhancement in the default mode network after stimulation in patients with prominent seizure reduction only, suggesting fMRI as a potential prognostic marker for tFUS effectiveness.

Though limited by the small sample size and the lack of a sham-control, reported seizure frequency reductions are promising. A transient decrement in memory functions was found in two of the studies and was discussed as a result of fatigue (Stern et al., 2021) or as a forced normalization phenomenon (Lee et al., 2022). Nonetheless, memory impairment due to medial temporal lobe sonication is conceivable and warrants careful consideration in prospective investigations. The three currently registered ongoing studies demonstrate a slight increase in scheduled patient number, yet only one RCT is listed (see Table 2 for details).

### 2.6 Disorders of consciousness

In a longitudinal open-label study, Lohse-Busch et al. (2014) examined the effect of global stimulation with a non-navigated TPS precursor in five patients that have been suffering from stable unresponsive wakefulness syndrome for 7–18 years. Patients received several treatment cycles, comprising of 12 sessions over 4 weeks, over a period of 2–4 years. After the treatment, clinically significant improvements in coma scales were noted. In four patients, non-verbal communication became feasible and in three patients the necessity for a feeding tube was eliminated.

In the framework of the first-in-man open-label clinical trial of thalamic tFUS, Monti and colleagues report several cases of patients with disorders of consciousness who were treated with 10 sonications targeted at the central thalamus. In the first case report, a patient with acute disorder of consciousness recovered from minimally conscious state 3 days after one stimulation session (Monti et al., 2016). A subsequent investigation in three patients with chronic minimal conscious state demonstrated improved responsiveness in two patients after two tFUS sessions (Cain et al., 2021). Finally, 11 patients with acute disorder of consciousness received one (n = 8) or two (n = 3) tFUS sessions with concurrent fMRI (Cain et al., 2022). Compared to the baseline, fMRI activation decreased in prefrontal areas, the anterior cingulate cortex, and the striatum during tFUS stimulation. While no benefit was found immediately after sonication, behavioral responsiveness was significantly enhanced the week following tFUS and was correlated with decreased functional connectivity of the targeted thalamus to frontal and subcortical areas.

The small, open-label studies report benefits in the patient’s responsiveness; however, spontaneous recovery particularly in acute stages of disorders of consciousness must be considered. By now, no ongoing or prospective study was found to be registered.

### 2.7 Other disorders

So far, only one clinical ultrasound neuromodulation study has been published in developmental disorders. Cheung et al. (2023b) investigated the effect of TPS in a randomized sham-controlled clinical trial in 32 participants between 12 and 17 years of age with autism spectrum disorder who received sonication directed towards the right temporoparietal junction. Autism symptoms and global clinical impression significantly improved in the verum group compared to sham, and changes sustained for at least 3 months. Besides this investigation in autism (NCT05408793, Table 2), attention deficit hyperactivation disorder is being investigated using TPS in an RCT (NCT05422274) and applying tFUS in an open-label study (NCT04497363).

Currently, the effectiveness of TPS in treating neurological Post-Covid-19 symptoms is under investigation in a sham-controlled RCT involving 120 individuals. Meanwhile, tFUS is being studied in a double-blind, non-randomized trial for stroke and an open-label study for age-related frailty.

## 3 Discussion

Despite the increasing volume of clinical research initiatives, non-invasive brain stimulation using ultrasound in neuropsychiatric conditions is still in its nascent stage. The majority of available literature consists of uncontrolled pilot trials or feasibility studies with limited sample size, and in some controlled trials an appropriate sham condition is missing. So far, randomized sham-controlled studies provide evidence of positive effects of ultrasound neuromodulation regarding cognitive enhancement in stroke, mood improvement in depression, increased motor cortex excitability in PD, as well as relief of schizophrenic and autistic symptoms. Although addressed in open-label studies only, findings regarding tremor attenuation, reduction in epileptic seizure frequency and emergence from chronic minimally conscious state after sonication are promising.

Several studies include objective markers of brain physiology change, such as functional imaging or EEG, providing insight about potential mechanisms of action of ultrasound neuromodulation. Depending on stimulation parameters, tFUS is supposed to act both excitatory and inhibitory. Zadeh et al. (2024) compared three different pulse repetition frequencies (PRF) with a constant duty cycle (DC) of 10% regarding their effect on corticospinal excitability and found attenuated MEP for 10 and 100 Hz but no change for 1,000 Hz compared to a sham condition. Zhang et al. (2023) investigated excitatory (PRF = 2000 Hz, DC = 40%) and inhibitory (PRF = 50 Hz, DC = 2%) TUS protocols using MEP and MR spectroscopy and found increased motor cortex excitability, decreased GABA concentration and increased Glx (glutamine + glutamate) concentration for the excitatory parameters. In the inhibitory stimulation protocol decreased MEP were found, along with substantially increased GABA concentration (19%) and significantly altered GABA/Glx ratio. These findings regarding differential inhibitory and excitatory TUS effects encourage applications to several neurological conditions needing (focal) upregulation, for example in neurodegenerative diseases like AD or PD, or suppression of neuronal activation as mandatory in epilepsy, for example. TPS was reported to induce long-term upregulation of functional activation and connectivity in AD (Beisteiner et al., 2019) and in healthy participants (Matt et al., 2022b), suggesting excitatory neuronal modulation with the potential to sustainably change brain networks and associated symptoms. However, TPS sonication parameters were only marginally varied up to now, leaving room for exploring inhibitory applications as well.

So far, both tFUS and TPS has proven to be well tolerated, without any notion of serious adverse events or morphological brain changes (Pasquinelli et al., 2019; Radjenovic et al., 2022). Rarely occurring side effects such as headache, mood changes, or fatigue were mild and transient.

Certainly, neurophysiological and clinical effects are highly dependent on specific sonication parameters. Regrettably, their consistent reporting is lacking in the existing literature, raising questions about which parameters should be prioritized and how they should be presented. In this rapidly changing field, it is mandatory to agree on common standards regarding reporting of methodological aspects, findings, adverse events, as well as recommendations for established and forthcoming clinical applications of TUS, which is currently under debate (Beisteiner et al., 2024; Martin et al., 2024). More randomized sham-controlled trials are needed to solidify the current evidence, preferentially with large cohorts (> 100 participants), comprehensive assessments of clinical, behavioral and neurophysiological changes, as well as longer follow-up periods to determine persistence of clinical changes.

## Author contributions

EM: Conceptualization, Data curation, Formal analysis, Investigation, Methodology, Project administration, Supervision, Validation, Writing – original draft, Writing – review & editing. SR: Data curation, Formal analysis, Investigation, Methodology, Validation, Writing – original draft, Writing – review & editing. MM: Conceptualization, Data curation, Formal analysis, Investigation, Methodology, Supervision, Validation, Writing – original draft, Writing – review & editing. RB: Conceptualization, Formal analysis, Funding acquisition, Methodology, Resources, Supervision, Validation, Writing – original draft, Writing – review & editing.

## References

[ref1] Badran B. W. Caulfield K. A. Stomberg-Firestein S. Summers P. M. Dowdle L. T. Savoca M.. (2020). Sonication of the anterior thalamus with MRI-guided transcranial focused ultrasound (tFUS) alters pain thresholds in healthy adults: a double-blind, sham-controlled study. Brain Stimul. 13, 1805–1812. doi: 10.1016/j.brs.2020.10.007, PMID: 33127579 PMC7888561

[ref2] Bancel T. Béranger B. Daniel M. Didier M. Santin M. Rachmilevitch I.. (2024). Sustained reduction of essential tremor with low-power non-thermal transcranial focused ultrasound stimulations in humans. Brain Stimul. 17, 636–647. doi: 10.1016/j.brs.2024.05.003, PMID: 38734066

[ref3] Bao S. Kim H. Shettigar N. B. Li Y. Lei Y. (2024). Personalized depth-specific neuromodulation of the human primary motor cortex via ultrasound. J. Physiol. 602, 933–948. doi: 10.1113/JP285613, PMID: 38358314

[ref4] Beisteiner R. Hallett M. Lozano A. M. (2023). Ultrasound neuromodulation as a new brain therapy. Adv. Sci. (Weinh) 10:e2205634. doi: 10.1002/advs.202205634, PMID: 36961104 PMC10190662

[ref5] Beisteiner R. Lozano A. M. (2020). Transcranial ultrasound innovations ready for broad clinical application. Adv. Sci. 7:2002026. doi: 10.1002/advs.202002026, PMID: 33304757 PMC7709976

[ref6] Beisteiner R. Lozano A. Di Lazzaro V. George M. S. Hallett M. (2024). Clinical recommendations for non-invasive ultrasound neuromodulation. SSRN Electron. J. doi: 10.2139/ssrn.4744451 39084519

[ref7] Beisteiner R. Matt E. Fan C. Baldysiak H. Schönfeld M. Philippi Novak T.. (2019). Transcranial pulse stimulation with ultrasound in Alzheimer’s disease—a new navigated focal brain therapy. Adv. Sci. 7:1902583. doi: 10.1002/advs.201902583, PMID: 32042569 PMC7001626

[ref8] Bobola M. S. Chen L. Ezeokeke C. K. Olmstead T. A. Nguyen C. Sahota A.. (2020). Transcranial focused ultrasound, pulsed at 40 Hz, activates microglia acutely and reduces Aβ load chronically, as demonstrated in vivo. Brain Stimul. 13, 1014–1023. doi: 10.1016/j.brs.2020.03.016, PMID: 32388044 PMC7308193

[ref9] Brinker S. T. Preiswerk F. White P. J. Mariano T. Y. McDannold N. J. Bubrick E. J. (2020). Focused ultrasound platform for investigating therapeutic neuromodulation across the human Hippocampus. Ultrasound Med. Biol. 46, 1270–1274. doi: 10.1016/j.ultrasmedbio.2020.01.007, PMID: 32088061 PMC7239323

[ref10] Bubrick E. J. McDannold N. J. Orozco J. Mariano T. Y. Rigolo L. Golby A. J.. (2024). Transcranial ultrasound neuromodulation for epilepsy: a pilot safety trial. Brain Stimul. 17, 7–9. doi: 10.1016/j.brs.2023.11.013, PMID: 38070706

[ref11] Cain J. A. Spivak N. M. Coetzee J. P. Crone J. S. Johnson M. A. Lutkenhoff E. S.. (2022). Ultrasonic deep brain neuromodulation in acute disorders of consciousness: a proof-of-concept. Brain Sci. 12:428. doi: 10.3390/brainsci12040428, PMID: 35447960 PMC9032970

[ref12] Cain J. A. Visagan S. Johnson M. A. Crone J. Blades R. Spivak N. M.. (2021). Real time and delayed effects of subcortical low intensity focused ultrasound. Sci. Rep. 11:6100. doi: 10.1038/s41598-021-85504-y, PMID: 33731821 PMC7969624

[ref13] Cheung T. Li T. M. H. Ho Y. S. Kranz G. Fong K. N. K. Leung S. F.. (2023a). Effects of transcranial pulse stimulation (TPS) on adults with symptoms of depression-a pilot randomized controlled trial. Int. J. Environ. Res. Public Health 20:2333. doi: 10.3390/ijerph20032333, PMID: 36767702 PMC9915638

[ref14] Cheung T. Li T. M. H. Lam J. Y. T. Fong K. H. Chiu L. Y. Ho Y. S.. (2023b). Effects of transcranial pulse stimulation on autism spectrum disorder: a double-blind, randomized, sham-controlled trial. Brain Commun. 5:fcad226. doi: 10.1093/braincomms/fcad226, PMID: 37701816 PMC10493640

[ref15] Cont C. Stute N. Galli A. Schulte C. Logmin K. Trenado C.. (2022). Retrospective real-world pilot data on transcranial pulse stimulation in mild to severe Alzheimer’s patients. Front. Neurol. 13:948204. doi: 10.3389/fneur.2022.948204, PMID: 36188380 PMC9515314

[ref16] Deveney C. M. Surya J. R. Haroon J. M. Mahdavi K. D. Hoffman K. R. Enemuo K. C.. (2024). Transcranial focused ultrasound for the treatment of tremor: a preliminary case series. Brain Stimul. 17, 35–38. doi: 10.1016/j.brs.2023.12.007, PMID: 38128826

[ref17] Dörl G. Matt E. Beisteiner R. (2022). Functional specificity of TPS brain stimulation effects in patients with Alzheimer’s disease: A follow-up fMRI analysis. Neurol. Ther. 11, 1391–1398. doi: 10.1007/s40120-022-00362-8 35633496 PMC9338196

[ref18] Eguchi K. Shindo T. Ito K. Ogata T. Kurosawa R. Kagaya Y.. (2018). Whole-brain low-intensity pulsed ultrasound therapy markedly improves cognitive dysfunctions in mouse models of dementia – crucial roles of endothelial nitric oxide synthase. Brain Stimul. 11, 959–973. doi: 10.1016/j.brs.2018.05.012, PMID: 29857968

[ref19] Fomenko A. Chen K.-H. S. Nankoo J.-F. Saravanamuttu J. Wang Y. El-Baba M.. (2020). Systematic examination of low-intensity ultrasound parameters on human motor cortex excitability and behavior. eLife 9:e54497. doi: 10.7554/eLife.54497, PMID: 33236981 PMC7728443

[ref20] Fong T. K. H. Cheung T. Ngan S. T. J. Tong K. Lui W. Y. V. Chan W. C.. (2023). Transcranial pulse stimulation in the treatment of mild neurocognitive disorders. Ann. Clin. Transl. Neurol. 10, 1885–1890. doi: 10.1002/acn3.51882, PMID: 37607114 PMC10578878

[ref21] Gibson B. C. Sanguinetti J. L. Badran B. W. Yu A. B. Klein E. P. Abbott C. C.. (2018). Increased excitability induced in the primary motor cortex by transcranial ultrasound stimulation. Front. Neurol. 9:1007. doi: 10.3389/fneur.2018.01007, PMID: 30546342 PMC6280333

[ref22] Grippe T. Shamli-Oghli Y. Darmani G. Nankoo J.-F. Raies N. Sarica C.. (2024). Plasticity-induced effects of Theta burst transcranial ultrasound stimulation in Parkinson’s disease. Mov. Disord. doi: 10.1002/mds.29836 38787806

[ref23] Hakimova H. Kim S. Chu K. Lee S. K. Jeong B. Jeon D. (2015). Ultrasound stimulation inhibits recurrent seizures and improves behavioral outcome in an experimental model of mesial temporal lobe epilepsy. Epilepsy Behav. 49, 26–32. doi: 10.1016/j.yebeh.2015.04.008, PMID: 25940106

[ref24] Hameroff S. Trakas M. Duffield C. Annabi E. Gerace M. B. Boyle P.. (2013). Transcranial ultrasound (TUS) effects on mental states: a pilot study. Brain Stimul. 6, 409–415. doi: 10.1016/j.brs.2012.05.002, PMID: 22664271

[ref25] Jeong H. Im J. J. Park J.-S. Na S.-H. Lee W. Yoo S.-S.. (2021). A pilot clinical study of low-intensity transcranial focused ultrasound in Alzheimer’s disease. Ultrasonography 40, 512–519. doi: 10.14366/usg.20138, PMID: 33730775 PMC8446491

[ref26] Jeong H. Song I.-U. Chung Y.-A. Park J.-S. Na S.-H. Im J. J.. (2022). Short-term efficacy of transcranial focused ultrasound to the Hippocampus in Alzheimer’s disease: a preliminary study. J. Pers. Med. 12:250. doi: 10.3390/jpm12020250, PMID: 35207738 PMC8878180

[ref27] Lee C.-C. Chou C.-C. Hsiao F.-J. Chen Y.-H. Lin C.-F. Chen C.-J.. (2022). Pilot study of focused ultrasound for drug-resistant epilepsy. Epilepsia 63, 162–175. doi: 10.1111/epi.17105, PMID: 34729772 PMC9297900

[ref28] Lee J. Kim Y. E. Lim J. Jo Y. Lee H. J. Jo Y. S.. (2024). Transcranial focused ultrasound stimulation in the infralimbic cortex facilitates extinction of conditioned fear in rats. Brain Stimul. 17, 405–412. doi: 10.1016/j.brs.2024.03.013, PMID: 38537689

[ref29] Legon W. Bansal P. Tyshynsky R. Ai L. Mueller J. K. (2018). Transcranial focused ultrasound neuromodulation of the human primary motor cortex. Sci. Rep. 8:10007. doi: 10.1038/s41598-018-28320-1, PMID: 29968768 PMC6030101

[ref30] Legon W. Sato T. F. Opitz A. Mueller J. Barbour A. Williams A.. (2014). Transcranial focused ultrasound modulates the activity of primary somatosensory cortex in humans. Nat. Neurosci. 17, 322–329. doi: 10.1038/nn.3620, PMID: 24413698

[ref31] Legon W. Strohman A. In A. Payne B. (2024). Noninvasive neuromodulation of subregions of the human insula differentially affect pain processing and heart-rate variability: a within-subjects pseudo-randomized trial. Pain. doi: 10.1097/j.pain.0000000000003171, PMID: 38314779 PMC11189760

[ref32] Leinenga G. Götz J. (2015). Scanning ultrasound removes amyloid-β and restores memory in an Alzheimer’s disease mouse model. Sci. Transl. Med. 7:278ra33. doi: 10.1126/scitranslmed.aaa2512, PMID: 25761889

[ref33] Lohse-Busch H. Reime U. Falland R. (2014). Symptomatic treatment of unresponsive wakefulness syndrome with transcranially focused extracorporeal shock waves. NeuroRehabilitation 35, 235–244. doi: 10.3233/NRE-141115, PMID: 24990026

[ref34] Mahdavi K. D. Jordan S. E. Jordan K. G. Rindner E. S. Haroon J. M. Habelhah B.. (2023). A pilot study of low-intensity focused ultrasound for treatment-resistant generalized anxiety disorder. J. Psychiatr. Res. 168, 125–132. doi: 10.1016/j.jpsychires.2023.10.039 39491902

[ref35] Martin E. Aubry J.-F. Schafer M. Verhagen L. Treeby B. Pauly K. B. (2024). ITRUSST consensus on standardised reporting for transcranial ultrasound stimulation. Brain Stimul. 17, 607–615. doi: 10.1016/j.brs.2024.04.013, PMID: 38670224 PMC12436198

[ref36] Matt E. Dörl G. Beisteiner R. (2022a). Transcranial pulse stimulation (TPS) improves depression in AD patients on state-of-the-art treatment. Alzheimer’s Dement. (New York) 8:e12245. doi: 10.1002/trc2.12245, PMID: 35169611 PMC8829892

[ref37] Matt E. Kaindl L. Tenk S. Egger A. Kolarova T. Karahasanović N.. (2022b). First evidence of long-term effects of transcranial pulse stimulation (TPS) on the human brain. J. Transl. Med. 20:26. doi: 10.1186/s12967-021-03222-5, PMID: 35033118 PMC8760674

[ref38] Meng Y. Pople C. B. Lea-Banks H. Abrahao A. Davidson B. Suppiah S.. (2019). Safety and efficacy of focused ultrasound induced blood-brain barrier opening, an integrative review of animal and human studies. J. Control. Release 309, 25–36. doi: 10.1016/j.jconrel.2019.07.023, PMID: 31326464

[ref39] Min B. K. Yang P. S. Bohlke M. Park S. Vago D. R. Maher T. J.. (2011). Focused ultrasound modulates the level of cortical neurotransmitters: potential as a new functional brain mapping technique. Int. J. Imaging Syst. Technol. 21, 232–240. doi: 10.1002/ima.20284

[ref40] Monti M. M. Schnakers C. Korb A. S. Bystritsky A. Vespa P. M. (2016). Non-invasive ultrasonic thalamic stimulation in disorders of consciousness after severe brain injury: a first-in-man report. Brain Stimul. 9, 940–941. doi: 10.1016/J.BRS.2016.07.008, PMID: 27567470

[ref41] Nicodemus N. E. Becerra S. Kuhn T. P. Packham H. R. Duncan J. Mahdavi K.. (2019). Focused transcranial ultrasound for treatment of neurodegenerative dementia. Alzheimer’s Dement. Transl. Res. Clin. Interv. 5, 374–381. doi: 10.1016/j.trci.2019.06.007, PMID: 31440580 PMC6700262

[ref42] Osou S. Radjenovic S. Bender L. Gaal M. Zettl A. Dörl G.. (2023). Novel ultrasound neuromodulation therapy with transcranial pulse stimulation (TPS) in Parkinson’s disease: a first retrospective analysis. J. Neurol. 271, 1462–1468. doi: 10.1007/s00415-023-12114-1, PMID: 38032371 PMC10896933

[ref43] Pasquinelli C. Hanson L. G. Siebner H. R. Lee H. J. Thielscher A. (2019). Safety of transcranial focused ultrasound stimulation: a systematic review of the state of knowledge from both human and animal studies. Brain Stimul. 12, 1367–1380. doi: 10.1016/j.brs.2019.07.024, PMID: 31401074

[ref44] Popescu T. Pernet C. Beisteiner R. (2021). Transcranial ultrasound pulse stimulation reduces cortical atrophy in Alzheimer’s patients: a follow-up study. Alzheimer’s Dement. Transl. Res. Clin. Interv. 7, 1–6. doi: 10.1002/trc2.12121, PMID: 33681449 PMC7906128

[ref45] Radjenovic S. Dörl G. Gaal M. Beisteiner R. (2022). Safety of clinical ultrasound neuromodulation. Brain Sci. 12:1277. doi: 10.3390/brainsci12101277, PMID: 36291211 PMC9599299

[ref46] Rezai A. R. Ranjan M. D’Haese P.-F. Haut M. W. Carpenter J. Najib U.. (2020). Noninvasive hippocampal blood-brain barrier opening in Alzheimer’s disease with focused ultrasound. Proc. Natl. Acad. Sci. USA 117, 9180–9182. doi: 10.1073/pnas.2002571117, PMID: 32284421 PMC7196825

[ref47] Reznik S. J. Sanguinetti J. L. Tyler W. J. Daft C. Allen J. J. B. (2020). A double-blind pilot study of transcranial ultrasound (TUS) as a five-day intervention: TUS mitigates worry among depressed participants. Neurol. Psychiatry Brain Res. 37, 60–66. doi: 10.1016/j.npbr.2020.06.004

[ref48] Riis T. S. Feldman D. A. Vonesh L. C. Brown J. R. Solzbacher D. Kubanek J.. (2023). Durable effects of deep brain ultrasonic neuromodulation on major depression: a case report. J. Med. Case Rep. 17:449. doi: 10.1186/s13256-023-04194-4, PMID: 37891643 PMC10612153

[ref49] Riis T. S. Losser A. J. Kassavetis P. Moretti P. Kubanek J. (2024). Noninvasive modulation of essential tremor with focused ultrasonic waves. J. Neural Eng. 21:016033. doi: 10.1088/1741-2552/ad27ef, PMID: 38335553

[ref50] Samuel N. Ding M. Y. R. Sarica C. Darmani G. Harmsen I. E. Grippe T.. (2023). Accelerated transcranial ultrasound neuromodulation in Parkinson’s disease: a pilot study. Mov. Disord. 38, 2209–2216. doi: 10.1002/mds.29622, PMID: 37811802

[ref51] Sanguinetti J. L. Hameroff S. Smith E. E. Sato T. Daft C. M. W. Tyler W. J.. (2020). Transcranial focused ultrasound to the right prefrontal cortex improves mood and alters functional connectivity in humans. Front. Hum. Neurosci. 14, 1–13. doi: 10.3389/fnhum.2020.00052, PMID: 32184714 PMC7058635

[ref52] Shimokawa H. Shindo T. Ishiki A. Tomita N. Ichijyo S. Watanabe T.. (2022). A pilot study of whole-brain low-intensity pulsed ultrasound therapy for early stage of Alzheimer’s disease (LIPUS-AD): a randomized, double-blind, placebo-controlled trial. Tohoku J. Exp. Med. 258, 167–175. doi: 10.1620/tjem.2022.J078, PMID: 36104179

[ref53] Shin D. H. Son S. Kim E. Y. (2023). Low-energy transcranial navigation-guided focused ultrasound for neuropathic pain: an exploratory study. Brain Sci. 13:1433. doi: 10.3390/brainsci13101433, PMID: 37891801 PMC10605299

[ref54] Shinzato G. T. Assone T. Sandler P. C. Pacheco-Barrios K. Fregni F. Radanovic M.. (2024). Non-invasive sound wave brain stimulation with transcranial pulse stimulation (TPS) improves neuropsychiatric symptoms in Alzheimer’s disease. Brain Stimul. 17, 413–415. doi: 10.1016/j.brs.2024.03.007, PMID: 38513821

[ref55] Stern J. M. Spivak N. M. Becerra S. A. Kuhn T. P. Korb A. S. Kronemyer D.. (2021). Safety of focused ultrasound neuromodulation in humans with temporal lobe epilepsy. Brain Stimul. 14, 1022–1031. doi: 10.1016/j.brs.2021.06.003, PMID: 34198105

[ref56] Truong D. Q. Thomas C. Hampstead B. M. Datta A. (2022). Comparison of transcranial focused ultrasound and transcranial pulse stimulation for neuromodulation: a computational study. Neuromodulation 25, 606–613. doi: 10.1016/j.neurom.2021.12.012, PMID: 35125300

[ref57] Wang Y. Li F. He M.-J. Chen S.-J. (2022). The effects and mechanisms of transcranial ultrasound stimulation combined with cognitive rehabilitation on post-stroke cognitive impairment. Neurol. Sci. 43, 4315–4321. doi: 10.1007/s10072-022-05906-2, PMID: 35141805

[ref58] Wang Z. Yan J. Wang X. Yuan Y. Li X. (2020). Transcranial ultrasound stimulation directly influences the cortical excitability of the motor cortex in parkinsonian mice. Mov. Disord. 35, 693–698. doi: 10.1002/mds.27952, PMID: 31829467

[ref59] Xu T. Lu X. Peng D. Wang G. Chen C. Liu W.. (2020). Ultrasonic stimulation of the brain to enhance the release of dopamine – a potential novel treatment for Parkinson’s disease. Ultrason. Sonochem. 63:104955. doi: 10.1016/j.ultsonch.2019.104955, PMID: 31945561

[ref60] Yi S.-S. Zou J.-J. Meng L. Chen H.-M. Hong Z.-Q. Liu X.-F.. (2022). Ultrasound stimulation of prefrontal cortex improves lipopolysaccharide-induced depressive-like behaviors in mice. Front. Psych. 13:864481. doi: 10.3389/fpsyt.2022.864481, PMID: 35573384 PMC9099414

[ref61] Zadeh A. Raghuram H. Shrestha S. Kibreab M. Kathol I. Martino D.. (2024). The effect of transcranial ultrasound pulse repetition frequency on sustained inhibition in the human primary motor cortex: A double-blind, Sham-Controlled Study. Brain Stimul 17, 476–484. doi: 10.1016/j.brs.2024.04.005 38621645

[ref62] Zhai Z. Ren L. Song Z. Xiang Q. Zhuo K. Zhang S.. (2023). The efficacy of low-intensity transcranial ultrasound stimulation on negative symptoms in schizophrenia: a double-blind, randomized sham-controlled study. Brain Stimul. 16, 790–792. doi: 10.1016/j.brs.2023.04.021 37121354

[ref63] Zhang T. Guo B. Zuo Z. Long X. Hu S. Li S.. (2023). Excitatory-inhibitory modulation of transcranial focus ultrasound stimulation on human motor cortex. CNS Neurosci. Ther. 29, 3829–3841. doi: 10.1111/cns.14303, PMID: 37309308 PMC10651987

[ref64] Zhang J. Zhou H. Yang J. Jia J. Niu L. Sun Z.. (2021). Low-intensity pulsed ultrasound ameliorates depression-like behaviors in a rat model of chronic unpredictable stress. CNS Neurosci. Ther. 27, 233–243. doi: 10.1111/cns.13463, PMID: 33112507 PMC7816209

[ref65] Zhou H. Niu L. Xia X. Lin Z. Liu X. Su M.. (2019). Wearable ultrasound improves motor function in an MPTP mouse model of Parkinson’s disease. I.E.E.E. Trans. Biomed. Eng. 66, 3006–3013. doi: 10.1109/TBME.2019.2899631, PMID: 30794160

